# Characteristics of owned dogs in rabies endemic KwaZulu-Natal province, South Africa

**DOI:** 10.1186/s12917-018-1604-z

**Published:** 2018-09-10

**Authors:** Melinda Hergert, Kevin Le Roux, Louis H. Nel

**Affiliations:** 1 Department of Paraclinical Sciences, Faculty of Veterinary Science, University of Pretoria, Onderstepoort, 0110 South Africa; 2 KwaZulu-Natal Department of Environment, Agriculture and Rural Development, Government Veterinary Services, 458 Townbush Road, Pietermaritzburg, 3202 South Africa; 3 Department of Biochemistry, Genetics and Microbiology, Faculty of Natural and Agricultural Sciences University of Pretoria, Pretoria, 0002 South Africa; 4 Global Alliance for Rabies Control, 529 Humboldt St., Suite 1, Manhattan, KS 66502 USA

**Keywords:** Canine, Rabies, South Africa, Population study

## Abstract

**Background:**

Canine rabies has been enzootic in the dog population of the KwaZulu-Natal province of South Africa since the mid-1970s and has been associated with high rates of human exposures and frequent transmissions to other domestic animal species. Several decades of control efforts, consisting primarily of mass vaccination programs, have previously failed to sufficiently curb rabies in the province. Despite this history of canine rabies, the target canine population has never been extensively studied or quantified. For efficient and effective vaccination campaign planning, the target population must be evaluated and understood. This study reports evaluated observations from survey records captured through a cross sectional observational study regarding canine populations and dog owners in rabies enzootic KwaZulu-Natal province, South Africa. The objective of this study was to aid government veterinary services in their current and ongoing efforts to eliminate canine rabies in the province by gaining information about the size and distribution of the owned dog population.

**Results:**

Thirty-eight percent of the households owned one or more dogs, with rural areas surveyed containing a significantly higher number of owned dogs than urban areas. The mean dog/person ratio for this study was 1:7.7 (range 1:5.4–1:31). The provincial sex ratio was 1.5:1 male to female, with the percentages for male dogs across the communities ranging from 53 to 61.5%. The age structure of this dog population indicates a high turnover rate. Dogs were kept mostly for guarding homes or livestock. Eighty-four percent of dogs had received a rabies vaccine at some point in their lifetime, almost all during a rabies campaign.

**Conclusions:**

The study indicates the majority of owned dogs can be handled by at least one member of the household, thus can be made readily accessible for rabies vaccination during a campaign. Characteristics of owned dogs in the province were similar to those studied in other African countries; however, there were remarkable differences in age, sex and husbandry practices compared to dogs in eastern or northern Africa. These geographical differences lend credence to the theory that canine populations are heterogeneous; therefore, target populations should be evaluated prior to intervention planning.

## Background

In developing countries the domestic dog (*Canis familiaris*) is both the principal reservoir and the primary vector for rabies in humans and other domestic animal species [1] and exposure to infectious dogs result in over 99% of human rabies cases worldwide [2–4]. In a recent study, this number was conservatively estimated at 59,000 cases per year [5], but the burden is likely to be higher, considering that half of the global human population lives in canine rabies endemic areas of the poverty stricken developing world. Canine (dog) rabies is endemic throughout Africa [6] and the continent carries an estimated 36.4% of the global burden of human rabies [5].

It is well established that the key to the prevention of human rabies, lies in the control of the disease in the canine reservoir [7, 8]. This objective is readily achievable through mass vaccination [3, 9]. However, to be truly effective, thorough knowledge of the characteristics of the canine population (including the true size of the population) is necessary for efficient planning and allocation of campaign resources [10, 11].

The World Health Organization (WHO) suggests that canine ecology surveys should be composed of two parts: one questionnaire for gathering information on the household, and another for collecting information on each dog owned by the household [12]. Household associated queries provide information about the member composition of the household, socioeconomics, religion, culture, number of owned dogs, dog bite histories, knowledge of rabies and other human related points of interest. The individual dog questionnaire gains descriptive statistics on each of the dogs owned by the household, as well as purpose, management and husbandry style. Non-dog owning community members can contribute with respect to the household questionnaire, thus gaining general information about the entire community. Area culture and infrastructure may affect the methodology employed in marketing and scheduling tactics of vaccination campaigns for rabies control. Central point vaccinations may be conducive to a township configuration, whereas rural areas may require house-to-house delivery of vaccine. Optimal frequency of vaccination campaigns which target at risk dog sub-populations, while considering economics and measurable outcomes are critical to the planning and sustainability of dog rabies control programs in developing countries [8, 13].

Two rabies virus variants are recognized in South Africa, respectively recognized in herpestids (mongooses, genets) and canines [14–17]. Historically, the largest percentage of human rabies cases in South Africa has occurred in KwaZulu-Natal (KZN) where canine variant is circulated and maintained within the large provincial population of domestic dogs [18–21]. Despite the long history of endemic canine rabies in KZN, extensive canine ecology studies have not been performed as part of the provincial disease control program and there is an inadequate understanding of the dog population – particularly in reference to population figures and ownership practices. Door-to-door mass rabies vaccination campaigns are conducted annually by KZN government veterinary services (GVS) with the cooperation of staff from the Departments of Health and Environmental Health using bright yellow trucks and loud hailer systems announcing their arrival. The objective of this study was to aid GVS in their current and ongoing efforts to eliminate canine rabies in the province by gaining information about the size and distribution of the owned dog population.

## Methods

### Study area and sampling procedure

**Fig. 1:**
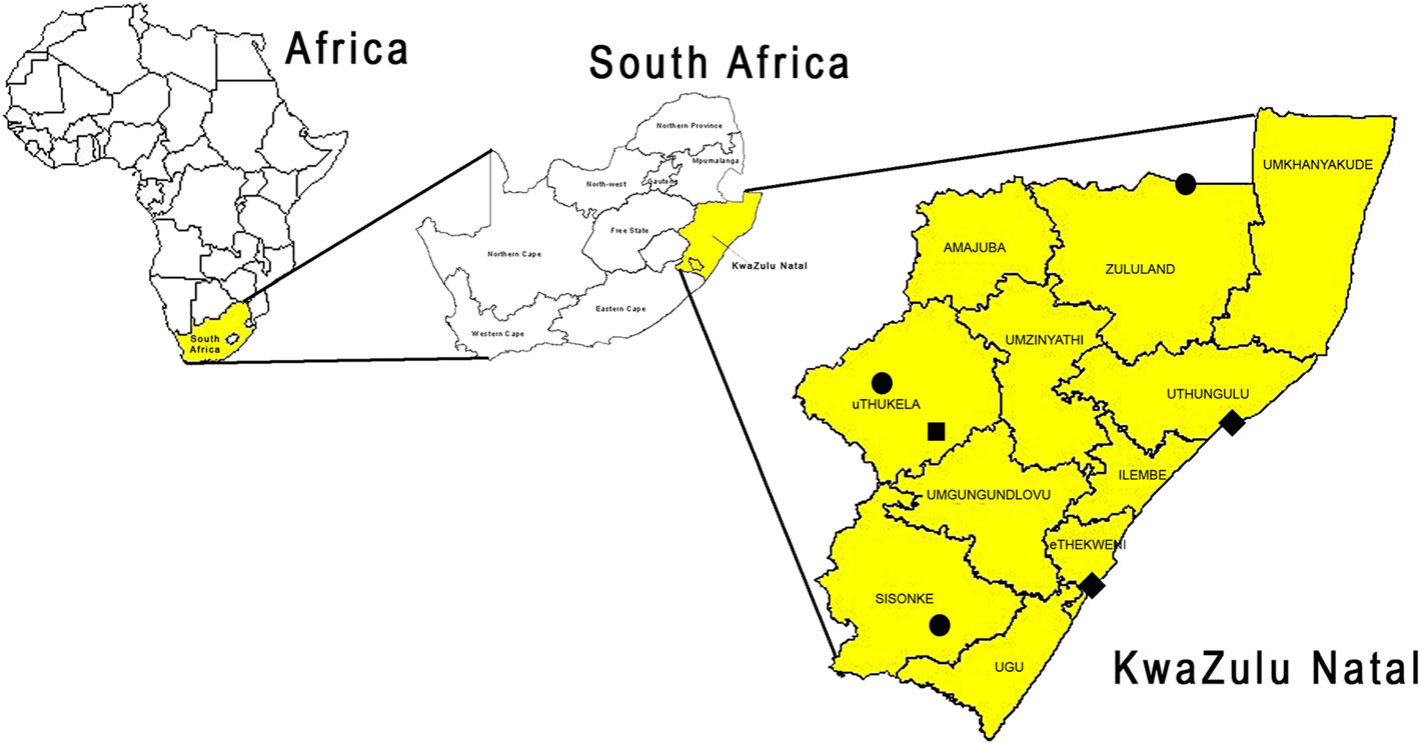
Geographical location of KwaZulu-Natal with the six study areas indicated. Note: Black square = Wembezi (peri-urban rabies free), black diamonds = Umlazi and Esikhawini (urban rabies enzootic), black circles = Ixopo, Pongola and St. Chads (rural rabies enzootic)

From September 2009 through January 2011, household surveys were conducted in six different communities across KZN province, covering three land use types (Fig. 1). Distribution of the 1992 households completing the surveys was 52% rural, 33% urban and 15% peri-urban. Rabies was enzootic in all areas, with the exception of the peri-urban community of Wembezi. Affluent urban and suburban areas where people keep dogs in confined spaces were excluded, as these dogs most likely have lower rabies risk due to fewer effective contacts between animals and access to private veterinary services [8]. Study areas were selected with the assistance of KZN DAERD GVS division, and consisted of high density townships and rural areas as described in Hergert et al. [22].

Simple random sampling and systematic surveys are difficult in developing countries due to logistical reasons; therefore, a cluster or ‘area’ design was used because rural area homesteads are not numbered, and informal housing settlements within townships are frequently haphazardly arranged [23, 24]. Clusters for this survey were identified using Google Earth maps at 4.6 km eye altitude with a grid in order to maintain consistent sampling methodology which allows for the random systematic selection of similar sampling units between differing geographical areas [10]. Clusters were numbered from left to right and selected by sampling with replacement using an online random number generator [22, 25]. There is potential for extrapolating survey results into the entire study area permitting generalizations providing that the geographical, socioeconomic and culture settings are the same or very close, which was true for each area type [10]. The desired minimum sample size for each area was 323 households.

### Questionnaire interviews

Utilizing WHO [26] canine rabies control guidelines, the questionnaires were composed of two parts; a household survey for collecting respondent demographics, and an individual dog survey for descriptive statistics of the owned dog population. The surveys were translated into isiZulu and then back translated to English. The surveys and interviewing methods were piloted in a township with comparable human demographics and a history of canine rabies. KZN DAERD Animal Health Technicians and students, Department of Health workers, Environmental Health workers and SPCA employees were trained to perform the surveys and acquire informed verbal consent from respondents. Only respondents who verbally agreed to the survey were interviewed. Any children between the ages of 14 and 18 years of age who answered the survey on behalf of the family had an adult family member present during the interview to provide verbal consent. All interviews were conducted on weekdays between the hours of 9 am and 3 pm.

### Data analysis

The data from each area was entered into a Microsoft Excel spreadsheet and then imported into SAS version 9.3 (SAS Institute, Inc., Cary, North Carolina, USA). Descriptive statistics were generated, and cross tabulations calculating Pearson’s Chi Square (χ^2^) were performed in tests of association. Area dog population numbers were calculated by multiplying the owned dog per person ratio by the most recent human census counts for the local municipality.

## Results

### Questionnaire interviews

A total of 1992 households consisting of 13,756 people (occupant range 1–34, median = 6) completed the surveys within the three targeted community types. Response rates ranged from 92 to 100% in the six areas surveyed. Survey questions were answered by a person defined as head of the household (68%, *n* = 1361), other adult (22%, *n* = 435), child over the age of 14 (9%, *n* = 183), and children under 14 with grandparent present (1%, *n* = 11). Category for respondent was missing in two cases. The sex of the respondent was not recorded.

Ninety-nine percent of the population was of Zulu culture. Eighty-four percent reported to be Christians, with 13% equally divided between Traditional African beliefs and the Shembe religion, a combination of Christianity and Zulu culture. Only two households reported to be of Muslim faith.

Eighty-four percent of houses were built of blocks or brick construction, with the remaining 16% being made of locally sourced materials like mud, manure, sticks or tin. Forty-three percent of households had a flush toilet, 52% had a pit latrine and 4% had no toilet facilities. Twenty-seven percent of houses had solid fences, most of which were found in urban communities. A pit near the house, or community dump was used for refuse removal by 55% of households surveyed. Twelve percent of the population surveyed owned cattle or goats, and 31% kept chickens. Fifty-five percent of households had at least one member working away from the home.

Thirty-eight percent (757/1992) of all of the households owned one or more dogs (range 1–19, median = 2). Individual descriptive statistics was attempted for every owned dog over suckling age; however, some records were missing substantial data points. Data were collected on 1667 individual dogs, of which 99 % were at home during some time of interview. The most common reason given (52%) for why a household did not own dogs was that they did not like them. Sixteen percent of households previously had dogs that died and wanted to replace them. Other reasons cited for not owning dogs were religion, lack of fencing, avoidance of conflict with neighbors and landlords, or that they could not afford them.

### Dog demographics

#### Age structure and sex ratios

Age structure of the owned dogs varied between areas. The rabies negative community of Wembezi reported the largest percentage of dogs three years or younger with a value of 79% (provincial average 66%). The largest age group in Wembezi was 1–2 years of age, which is suggested a high population turnover. The two urban townships showed the greatest disparity in range of elderly dogs. The urban township of Umlazi had the highest percentage of dogs over the age of five years at 29%, while the urban township of Esikhawini had the lowest percentage of dogs older than five years with a value of 13.7%. Of the thirty respondents who did not know the age of the dog, seventeen were not the owner. Where dog owners could recall the lifespan of dogs they no longer had, the average lifespan was reported as 4.9 to 6.5 years across the areas surveyed.

The provincial sex ratio was 1.5:1 male to female, with the percentages for male dogs across the communities ranging from 53 to 61.5%. Lactating females with litters of puppies present were most commonly seen in the rural areas. Geographically, there was no significance between sex of the dog and area surveyed, determined by the Chi Square test for independence (Chi Square = 1.8917, DF = 5, *p* = 0.86, Cramer’s V = 0.034).

#### Reproductive history of female dogs

Of the breeding age females (*n* = 293), 46% had whelped previously. Ninety-three percent (272/293) had given birth within the last 12 months. Data on litter size were missing for 21 cases and disposition from one case. Based upon owner recollection there were 1239 pups born in 251 litters giving an average litter size of 4.9 pups per litter (range 1–14). From these litters 32% of pups died before weaning age, 9% were sold, 26% were given away, and 32% remained in the original household. One litter was reported as stolen and another had been killed by the bitch. No owners reported killing or abandoning the puppies. Of the 25 litters that were sold, 48% were born to bitches that had been bought and 44% from bitches that were received as gifts (Table 1). Only two of the litters born to bitches reported as being kept for breeding were sold, and two of the litters were reported as having died. It is unknown if any puppy deaths were husbandry related, including exposure to elements, insufficient diet, overwhelming parasites, or infectious diseases (ex. Parvo, distemper, canine herpes, or *Brucella canis*). None of the litters from the hunting bitches were reported as sold. The breed of the dogs was not recorded because overwhelmingly, the community’s dogs were of the Africanis or ‘local’ land race [27, 28].

**Table 1:** Cross-tabulation of disposition of 271 litters of pups with the purpose of the bitch in surveyed communities KZN September 2009 – January 2011

Disposition of Pups	Purpose of Dog						Total
	Breeding	Guard house	Guard livestock	Hunting	Other	Pet	
Died	2	73	5	4	2	1	87
Gave away	1	58	1	4	1	4	69
Bitch killed	0	1	0	0	0	0	1
Sold	2	21	0	0	0	2	25
Still have	5	73	4	5	0	1	88
Stolen	0	1	0	0	0	0	1
Total	10	227	10	13	3	8	271

#### Desire and value of dog sterilization

Out of the 1794 total dogs observed, only 7 (5 males, 2 females) were reported as having been sterilized. One dog from the urban community of Umlazi had been sourced from the SPCA where adoption policy dictates that animals must be sterilized prior to leaving the shelter. As population turnover is an important characteristic in dog ecology and rabies control, respondents were asked if they would like to have their dogs sterilized to which 60 % (944/1552) responded positively. Odds ratio calculation (1.097) showed no influence or protective factor regarding sex of dog and owner desire for sterilization. Dog owners were further asked what monetary value they would place on sterilization surgery. There were four categorical responses to the question ranging from 0 to &gt; 100 South African Rand (SAR). Less than 7% of respondents placed a value on this service of &gt; 50 SAR (Table 2). The categories of 51–100 SAR and &gt; 100 SAR were collapsed into the category of &gt; 50 SAR for analysis and reporting.

**Table 2:** Owner valuation of sterilization services in South African Rand (SAR)

	Percent Owner Valuation of Veterinary Sterilization Service		
	*0 SAR*	*1–50 SAR*	*&gt; 50 SAR*
Ixopo	42	56	2
Pongola	69	26	5
St. Chad’s	59	38.5	2
Umlazi	49	40	11
Esikhawini	57	33	10
Wembezi	62	31	7
Provincial Average	56.33%	37.42%	6.17%

#### Dog densities and population estimates

Rural areas surveyed contain a significantly higher number of owned dogs than urban areas. Dog owning households varied from 45 to 64% in the three rural communities versus 12–15% in the two urban communities (Table 3). The peri-urban community of Wembezi is interesting in that it parallels human densities seen in urban areas with an average of six persons per household; whereas the dog statistics are more similar to the rural communities with 39% of households owning dogs and a dog/human ratio of 1:6. The mean dog/person ratio for this study was 1:7.7 (range 1:5.4–1:31). Area dog population numbers were calculated based upon the most recent human census counts for the local municipality [29, 30].

**Table 3:** Dog densities calculated from six surveyed communities KZN 2009–2011

	Rural			Urban		Peri-urban
	St. Chad’s	Ixopo	Pongola	Umlazi	Esikhawini	Wembezi
^a^HH Interviewed	357	342	346	334	318	300
%^a^DOHH	52%	64%	45%	12%	15%	39%
Persons/HH	7.42	7.4	8.56	6.34	5.54	5.91
Total Dogs	467	473	412	69	77	296
Dogs/HH	1.31	1.39	1.19	0.21	0.24	0.99
Dogs/DOHH	2.54	2.16	2.64	1.68	1.64	2.55
Dogs/Person	1:5.7	1:5.4	1:7.2	1:31	1:23	1:6
Dogs/Km^2^	276	9.2	5.2	476	495	324
Estimated Dog Population	2159^c^	1112^c^ 14,982^b^	17031^b^	24,194^c^	3086^c^	2916^c^
Male dogs	261	267	217	37	37	125
Female dogs	170	180	155	28	30	78
Sex ratio M:F	1.5	1.5	1.4	1.3	1.2	1.6

^a^*HH* Household, *DOHH* Dog owning household; ^b^Dog populations calculated for municipality; ^c^Dog populations calculated for local area

#### Dog management and husbandry

Dog management and husbandry comprises many variables including food source and frequency, handling of the dog, dog purpose, level of restriction, and provision of shelter. Most of the owned dogs were reported as guard dogs (87%), either for the homestead (85%) or livestock (2%). Less than 5% were considered as pets. Three percent of dogs were used for breeding and 5% for hunting. Only 17% of the dogs in the survey were reported as being fully restricted to the household by a chain or tether. Twenty percent of dogs were restricted for at least part of the day. The remaining 63% were never restricted and allowed to roam at will. Twenty-three percent of the dogs wore collars regardless of their level of restriction. Some type of shelter was provided for 59% of the dogs.

Over 94% of dog owners believed that they provided enough food for their dogs. Nine respondents admitted they did not feed their dogs every day. Although 19% of owners reported purchasing commercial dog food for their animals, the majority of owned dogs (73%) were fed left-over human food from the household. The remaining dogs were left to scavenge from rubbish pits or given butcher’s waste. Thirty-four percent of respondents said they fed dogs that they did not own on their property. Of those, 65% knew these dogs which were scavenging meals at their house; the remaining 35% were unknown dogs but were not considered strays by the respondents. No dogs were identified by respondents as community owned, neighborhood or stray dogs.

Seventy-two percent of the dogs were reported as owned by the head of the household. The remaining dogs were owned by other adult males or females (18%), the household children (7%), or shared ownership by everyone in the household (3%). Twenty-two percent of dogs could only be handled by their owner, 11 % could be handled by the children, and 65 % could be handled by everyone in the household. Less than 2% of dogs were reported as unmanageable.

Most of the dogs were either bought (44%) or received as gifts (38%). Seventeen percent of dogs appeared to be locally sourced, as they had come either from a neighbor or from the owners own bitch. Less than 2% of dogs had come to the current residence with a family member who had moved from another location, and only one dog was reported as adopted from the SPCA.

#### Rabies vaccination status of dogs

The individual dog survey allowed for the collection of the rabies vaccination status of each dog over suckling age in the household; however, rabies data points were missing for 47 of the 1667 animals. Eighty-four percent (1361/1620) of dogs with complete records had received a rabies vaccine at some point in their lifetime, and almost all of these dogs had last been vaccinated by an animal health technician during a rabies campaign. Less than 2% of dog owners reported that their dog had been vaccinated by a private veterinarian. Sixty-four percent (1043/1620) of the dogs were reported as having been immunized within the last one year. Owners said they could provide proof of vaccination cards in 82% of cases.

Respondents who had not presented their dogs at the last government vaccination campaign were asked why. Lack of owner awareness and campaign timing contributed the most to this failure (*n* = 137, 61.5%). Extraneous reasons (*n* = 85, 38.5%) commonly given for why dog owners said they had not presented their dogs for vaccination were: dog new to the household, dog thought to be too young for vaccination, or the dog had run away from the technicians. Respondents who said they did not want the vaccine were not queried further as to why.

#### Rabies vaccination status in cats

From 2001 to 2011, 539 cat brain samples were submitted to the provincial laboratory, with only thirty positive results by fluorescent antibody test [KZNDAERD personal communications 2012]. Seventeen percent of households surveyed owned a total of 542 cats (range of 1–10, median = 1). The ratio of dogs to cats in KZN surveyed households was 3.3:1. Sixty-three percent (*n* = 342) of the owned cats were reported as vaccinated against rabies, of which 226 (66%) were from homes where the dogs had been rabies vaccinated during the most recent government campaign. Cat owners were not asked for proof of vaccination.

### Discussion

The personal interview method employed for conducting these door-to-door surveys produced a high return rate of 98% for this study. Developing countries frequently do not have infrastructure for landline telephone systems and mail delivery for every eligible household; therefore, home-based interviews are most suitable for data collection [31, 32].

Rural populations surveyed owned considerably more dogs than the urban and peri-urban communities. Dog owning households varied from 45 to 65% in the three rural communities versus 12–15% in the two urban communities. Similar findings were reported from Zambia where 11% of households in urban Mutendere owned dogs and 42% in rural Palabana kept dogs [33]. Reports from urban Tanzania show 13% of households kept dogs [34]. In rural Sri Lanka, 57% of surveyed households owned dogs [35]. In contrast, Oboegbulem and Nwakonobi [36] found that 38.2% of urban households in Lagos, Nigeria owned dogs, compared to 20% of rural households. Fifty-two percent of households that did not own dogs in the surveys said it was because they did not like them. This consideration is unlike results from Kenya where only 7% of respondents said they did not like dogs [13]. Other reasons provided for not owning dogs included avoiding conflict with landlords and neighbors, and not having a fence to contain the dog. Fear of reprisal from neighbors, and not having fences were also common reasons for not owning dogs by Zambian residents [33].

The mean dog/person ratio for this study was 1:7.7 (range 1:5.4–1:31), generally similar to ratios from rural (1:6.7) and urban (1:45) Zambia reported by De Balogh et al. [33]. Dog densities measured in this study varied from 6.3 to 495 dogs per square kilometer, with highest densities seen in the urban townships. The estimated national averages for Zimbabwe were 1:6.5 dogs/person and 3.4 dogs/km^2^ [31]. In rural Kenya, dog densities were estimated at 6 to 21/km^2^ [13]. Urban Tanzania reported dog densities as 1:14 dogs/person and 334 dogs/km^2^ [34]. Densities from 30 dogs/km^2^ up to 3000/km^2^ have been reported for rural and urban/suburban Sri Lanka respectively with human dog ratios of 1:7 to 1:16 [35, 37]. Beran and Frith [38] concur with large urban dog densities when they reported 681 to 2388 dogs/km^2^ from the city of Guayaquil, Ecuador. However, the dog to human ratio for Guayaquil (1:7.2) was more comparable to rural areas rather than urban centers of Africa, which was a similar finding to the unique peri-urban area of Wembezi in this study. The high dog densities of urban areas correspond to high human densities in urban areas, and as dogs are dependent upon humans directly or indirectly for their food resources the dog population will grow with human populations as long as the people remain tolerant of the dogs [39, 40]. Western countries generally report dog/human ratios of 1:6 to 1:10 [37]. Despite some similarities in the African countries studied these variations in dog densities clearly point to the heterogeneity of dog populations across the world. Compared to Latin American and Asian examples, lower dog densities and human:dog ratios prevail in the African countries where such studies were done.

High dog densities in urban areas suggest the probability of high effective contact rates between dogs; however, solid fencing was found in over 40% of urban households visited in KZN. Fencing was seen at less than 16% of households in rural areas. In contrast, only 9% of houses in urban Mutendere, Zambia had solid fences [33] and only one household in the communal lands of Zimbabwe had a dog-restricting fence [40]. In our study, over half of the surveyed households utilize a pit by the house for rubbish removal, which can be an attractant for roaming dogs. In urban areas with municipal services respondents frequently commented that dogs were disturbing rubbish bins; a similar finding by McCrindle et al. [41] from the Johannesburg township of Soweto. The quantity of random dump-sites on roadsides and discarded litter in urban townships provides resources for scavenging dogs. Rural area roadsides and pathways were cleaner despite the utilization of open pits by homes. Though none of the dogs in the study were referred to as neighborhood or community dogs by respondents, many free-ranging dogs were purposely fed on non-owners’ property suggesting communal care. In Zimbabwe, 78.6% of households reported foreign dogs scavenging in the open pits containing human-derived food waste on their property [40]. It is possible that many scavenging dogs in this study were owned since 62% of owned dogs were allowed to roam for scavenging and socialization at all times. Over 70% of the owned dogs in the Eastern Cape Province of South Africa and 79% of owned dogs in Antananarivo, Madagascar were reported as being allowed to roam freely [42, 43]. Only 17% of KZN dogs were reported as always restricted to the yard and another 20% were only chained for part of the day. Many owners reported they let their dogs off the chain to roam and scavenge at night, which was also described from Lagos, Nigeria [36]. Over 53% of houses in Zimbabwe do not have toilets, allowing for the scavenging of human waste by roaming dogs [40]. Some respondents in this survey said they were afraid to visit the pit toilet at night for fear of the scavenging dogs.

For safety and logistical reasons, we relied on respondent knowledge to gain age data on the owned dogs. We found that more than 60% of the owned dog population was 3 years of age or younger, indicating a high population turnover. Similar numbers were reported from a local municipality in the Eastern Cape Province where they found 64% of the dogs were 3 years of age or younger [42]. This is close to the mean age of 2.6 years found by in rural Bophuthatswana, South Africa [44], with 2.8 years reported from Kenya [13] and 2.6 years from the Thungsong District of Thailand [45]. Canine ecology studies conducted in Zimbabwe, Zambia and Tanzania have reported a lower median age for the dog population of 1.9 to 2.2 years [31, 33, 34, 40, 46]. Dogs in rural Sri Lanka had a higher mean age of 3.5 years, with the male dogs living slightly longer, 3.7 years, compared to females at 3.1 years [35]. Brooks [31] also reported male dogs in Zimbabwe living six months longer than female dogs. Dog owners in this survey estimated the average lifespan for their dogs to be 5.5 years, a year older than was estimated for dogs in Zimbabwe [31].

Historically sex ratios in recorded dog populations of Africa are skewed towards males [32, 34, 40, 43]. As seen in other canine studies, this study showed that male dogs out number females with a sex ratio of 1.5:1. This is in close agreement with the Eastern Cape Province of South Africa where the ratio was 1.7:1 as well as urban Madagascar 1.5:1 [42, 43]. Tanzania and Zimbabwe have reported ratios of 1.3:1 [31, 34]. The Thungsong District of Thailand revealed a 2:1 M:F ratio in the owned dog population [45]. The Mirigama area of Sri Lanka also had a preponderance of male dogs (73.6%) [35]. In Kenya, Kitala et al. [13] found that male members of the household believed that male dogs make better guard dogs and hunters, thus the tendency to provide better husbandry practices for male dogs. Male dogs are the preferred choice for guard dogs in urban United States [47]. Residents of all socioeconomic classes in Guayaquil, Ecuador preferred male dogs 56 to 68% [38]. Results showing more male dogs than females in all age brackets are consistent with findings from other countries except Nigeria where a sex ratio biased towards females in both rural (0.8:1) and urban (0.9:1) areas was reported [36]. Perhaps the fact that the head of the household, or males in general, more frequently own the dogs, leads to this tendency for male favored sex bias. The select killing of female pups in Tunisia creates a skewed sex ratio that may be more about population control than sex preference [26].

Controlling reproduction in owned dogs is not a wide spread practice, as only seven dogs in the survey were reported as castrated or spayed. Although over 60% of dog owners said they would like to have their dogs sterilized, 57 % said they would not consider paying for the surgery. Respondents either did not value the service or could not comprehend the associated costs. Ninety-three percent of the breeding age females were reported to have whelped within the last 12 months. In Kenya and Zambia, 85% and 83% of reproductive age bitches had whelped in the last year [13, 33]. In contrast, nearly 64% of female dogs in Guayaquil over the age of one year had never whelped and only 50% of owned females in Antananarivo had been pregnant in the last year [38, 43].

In KZN, 1239 pups were born to 251 litters giving an average litter size for the province of 4.9 puppies per litter with a range of 1 to 14 across the six communities. Thirty-two percent of puppies born to bitches in this survey died prior to weaning age, which is similar to 38% seen in Guayaquil [38]. This figure is higher than the 22% reported from Kenya, but less than the 53% pre-weaning mortality of communal Zimbabwe [13, 40]. De Balogh et al. [33] reported from Zambia that over 50% of the puppies born died within a year, while nearly 30% had been given away or sold, leaving less than 20% remaining in households. In this study 35% of puppies were either sold or given away, while 32% remained in the household. One owner reported his litter had been stolen, while another reported that the bitch had killed the pups. No respondents reported killing or abandoning the pups unlike in Tunisia where 61% of unwanted puppies were killed as a form of population control [26].

As less than 10 % of the puppies were sold, the question arises of why some dogs are considered worthy of purchase. Of the 25 litters that were sold, 48% were born of bitches that had been bought and 44% from bitches that were received as gifts. Although in some communities greyhound type dogs were seen, a comment cannot be made about if the dogs reported as breeders were of pure bred origin. It would be expected that dogs of pure bred origin would be more valuable monetarily, thus the puppies from these litters would be kept or sold rather than given away. In this study, most litters from bitches reported as being kept for breeding were not sold. None of the litters were sold from hunting bitches. Of all of the litters reported as sold, 84% came from bitches whose purpose was to guard the house.

Over 80% of dogs in KZN were either purchased or acquired as gifts. Unlike dogs from the Machakos District of Kenya where Kitala et al. [13] found 35% of dogs originated within the household, less than 12% of KZN dogs came from household bitches. Despite increased human mobility via taxis and buses, less than 2% of the dogs came to the present household with a family member who moved. In Guayaquil, dogs appear to migrate more as Beran and Frith [38] report that 100 to 200 dogs (~ 26% of the population) were brought into the city daily being presented as gifts or with people moving. Despite some respondent complaints about dogs in the community, 16% of households that did not own dogs wanted to get dogs. This desire for dogs was commonly recorded by Kitala et al. [13] in Kenya and by Butler and Bingham [40] in Zimbabwean communities.

There appears to be little difference in the general husbandry and management of dogs between the three community types surveyed. The urban area of Umlazi reported the most dogs kept as pets at 18.5%, with the other urban are of Esikhawini and peri-urban Wembezi trailing at 12% and 9% respectively. The majority of owned dogs in KZN are kept to guard homes or livestock (87%). Less than 5% of dogs in rural areas were kept as pets, and dogs used for hunting were uncommon (4%) as was also reported by Butler [48] in Zimbabwean communal lands. Unlike in Nigeria, no dogs were reported as used for a meat source or for scavenging the homestead in this study [36, 49]. In Guayaquil, Ecuador 70% of canines were kept as guard dogs and 17.9% were kept as mascots or pets [38]. The role of guard dog corresponds with findings in other developing countries including Zimbabwe reporting 60% guard dogs [31, 40], Kenya over 99% guard dogs [13], Antananarivo reported 81% [43] and Thailand with 83% guard dogs [45]. Although the majority of dogs in Thailand are meant to guard the house, only 56% were fed food from the resident household, few were provided shelter and most are permitted to roam freely during the day.

Dog owners in KZN report that they feed their dogs adequately. Observations of owned dogs and dogs roaming streets showed them to be in reasonable body condition as was also reported from Antananarivo [43]. Dogs in rural areas were primarily fed left over human food from the household. In urban Umlazi nearly 70% of dogs were fed commercial dog food. Although the other urban and peri-urban communities also reported feeding commercial food, more than 50% of the dog’s diet was left over human food. Dogs in communal lands of Zimbabwe were fed maize meal porridge at least once a day and sometimes milk or bones [40]. Dogs in confinement in urban settings of Nigeria were more likely to directly receive food from their owners, whereas dogs in rural areas that were allowed to roam were somewhat self sufficient, being irregularly fed by their owners and scavenging the neighborhood for the remainder of their needs [36]. Dogs in Madagascar were fed with family food 81%, or a commercial diet 12%, and 7.1% were not fed by their owners [43]. Only nine respondents from this study stated that they did not feed their dogs and left them to scavenge. Kitala et al. [32] reported only 5 % of dogs being fed commercial dog food and the remainder fed on household leftovers and waste.

Accessibility of dogs for vaccination should not be an issue for KZN, as 76% of the dogs were reported as being able to be handled either by everyone in the household or at least the children. GVS should be able to vaccinate these dogs during a campaign provided a family member who can handle the dogs is home during the campaign. De Balogh et al. [33] reported good accessibility of dogs for parenteral vaccination in Zambia at central point locations as children could locate and present the dogs for vaccination. Although 98.6% of the owned dogs in this survey were at home during the visit, many owners reported their dogs would run away once the vaccinations begin. Door-to-door campaigns are currently conducted in KZN, which was also the suggested method for larger settlements in Zambia, especially for those households with many dogs [33].

The study indicates that most of the owned dog population of KZN is adequately rabies vaccinated with coverage of 74% using a vaccine that is labeled with three-year duration of immunity. However, there were two areas, rural Pongola (62%) and urban Umlazi (57%), with coverage levels under the recommended 70% [10, 22]. Thirty-five percent of the dogs in Antananarivo were reported as vaccinated against rabies, but only 7.2% had a valid certificate or vaccination card [43]. In Thungsong District of Thailand, 70% of the owned dog population had been vaccinated in the last 6 months [45]. For some of the areas in this survey, vaccination campaigns had not been conducted for over one year.

## Conclusion

The response rate from this survey showed that household surveys are an effective tool for gathering information about communities and the dogs they own. Government vaccination campaigns should be continued in KZN, as most of the dogs and cats in the study had been previously vaccinated by GVS. In order to increase rabies vaccination coverage, consideration should be given to holding vaccine campaigns over many days with varied hours and possibly on weekends in the high risk urban area of Umlazi where many dog owners work away from the household. Although most dogs in KZN are allowed to roam at will, they can be handled by most household members and can be made readily accessible for rabies vaccination during a campaign. Evidence that most of the dogs across the province were left to roam freely and appear to have a high population turnover and inadequate vaccination coverage in high risk areas might help explain why canine rabies persists despite current methods employed by veterinary services. Population characteristics of the dogs in this survey are similar to dogs studied in other southern African countries; however, there were notable differences in age, sex and husbandry compared to dogs in eastern or northern Africa. Populations of stray or unowned dogs cannot be properly assessed through the use of household questionnaires, and our study focused solely on owned dogs, which is a limitation of the study. While it appears that stray dogs are few in this province, data was not gathered to substantiate this claim.

## Abbreviations

DAERD: Department of Agriculture, Environment, and Rural Development
GVS: Government Veterinary Services
KZN: KwaZulu-Natal
SAR: South African Rand
WHO: World Health Organization
